# Increased activated memory B-cells in the peripheral blood of patients with erythema nodosum leprosum reactions

**DOI:** 10.1371/journal.pntd.0006121

**Published:** 2017-12-18

**Authors:** Edessa Negera, Stephen L. Walker, Yonas Bekele, Hazel M. Dockrell, Diana N. Lockwood

**Affiliations:** 1 London School of Hygiene and Tropical Medicine (LSHTM), Faculty of Infectious Tropical Diseases, London, United Kingdom; 2 Armauer Hansen Research Institute (AHRI), Addis Ababa, Ethiopia; Institut Pasteur, FRANCE

## Abstract

B-cells, in addition to antibody secretion, have emerged increasingly as effector and immunoregulatory cells in several chronic inflammatory diseases. Although Erythema Nodosum Leprosum (ENL) is an inflammatory complication of leprosy, the role of B- cell subsets has never been studied in this patient group. Therefore, it would be interesting to examine the contribution of B-cells in the pathogenesis of ENL. A case-control study design was used to recruit 30 untreated patients with ENL and 30 non-reactional lepromatous leprosy (LL) patient controls at ALERT Hospital, Ethiopia. Peripheral blood samples were obtained before, during and after treatment from each patient. Peripheral blood mononuclear cells (PBMCs) were isolated and used for immunophenotyping of B- cell subsets by flow cytometry. The kinetics of B-cells in patients with ENL before, during and after Prednisolone treatment of ENL was compared with LL patient controls as well as within ENL group. Total B-cells, mature B-cells and resting memory B-cells were not significantly different between patients with ENL reactions and LL controls before treatment. Interestingly, while the percentage of naive B-cells was significantly lower in untreated ENL patients than in LL patient controls, the percentage of activated memory B-cells was significantly higher in these untreated ENL patients than in LL controls. On the other hand, the percentage of tissue-like memory B-cells was considerably low in untreated ENL patients compared to LL controls. It appears that the lower frequency of tissue-like memory B-cells in untreated ENL could promote the B-cell/T-cell interaction in these patients through downregulation of inhibitory molecules unlike in LL patients. Conversely, the increased production of activated memory B-cells in ENL patients could imply the scale up of immune activation through antigen presentation to T-cells. However, the generation and differential function of these memory B-cells need further investigation. The finding of increased percentage of activated memory B-cells in untreated patients with ENL reactions suggests the association of these cells with the ENL pathology. The mechanism by which inflammatory reactions like ENL affecting these memory cells and contributing to the disease pathology is an interesting area to be explored for and could lead to the development of novel and highly efficacious drug for ENL treatment.

## Author summary

Some leprosy patients develop reactions which cause a significant morbidity and mortality in leprosy patients. There are two types of leprosy reactions, type 1 and type 2 reactions. Type 2 or Erythema nodosum leprosum (ENL) is an immune-mediated inflammatory complication of leprosy which occurs in lepromatous and borderline lepromatous leprosy patients. The exact cause of ENL is unknown. Immune-complexes and T-cells are suggested as the aetiology of ENL. However, the contribution of B-cells in ENL reactions has never been addressed. In the present study we described the role of B-cell subsets in ENL reaction and compared with non reactional LL patient controls before, during and after corticosteroids treatment. We found increased antigen experienced and activated B-cells in untreated ENL patients compared to those without the reaction (LL patients). This implies that B-cells are associated with ENL pathology. Therefore, the finding provides a ground for future research targeting B-cells to develop effective drug for ENL treatment.

## Introduction

B-cells enable the antigen-specific humoral immunity by forming highly specific antibodies during primary immune response. B-cells within the lymphoid tissue of the body such as bone marrow, spleen and lymph nodes, are stimulated by antigenic substances to proliferate and transform into plasma cells and the plasma cells in turn produce immunoglobulins which bind to cognate antigen [1]. Although B-cells are traditionally known as precursors for antibody-secreting plasma cells, they may also act as antigen-presenting cells (APC) and play an important role in the initiation and regulation of T and B cell responses [1, 2]. However, B-cells may also involve in disease pathology especially in autoimmune disorders. The pathogenic roles of B-cells in autoimmune diseases occur through several mechanistic pathways that include autoantibodies, immune-complexes, dendritic and T-cell activation, cytokine synthesis, chemokine-mediated functions, and ectopic neolymphogenesis [2].

Memory B-cells are B-cell sub-types that are formed within the germinal centres following primary infection and are important in generating an accelerated and more robust antibody-mediated immune response in the case of re-infection also known as a secondary immune response. Recent advances in tracking antigen-experienced memory B-cells have shown the existence of different classes of memory B-cells that have considerable functional differences. Currently there are three types of memory B-cells: resting, activated and tissue like memory B-cells, [3]. Activated memory B-cells have been shown to function as effective antigen presenting cells (APCs) to naive T-cells [4]. Tissue-like memory B-cells (TLM) expressed patterns of homing and inhibitory receptors similar to those described for antigen-specific T-cell exhaustion. Tissue like memory B-cells proliferate poorly in response to B-cell stimuli, which is consistent with high-level expression of multiple inhibitory receptors. Higher percentage of TLM has been reported in immunosuppressive diseases such as HIV [5, 6].

Leprosy is a spectrum disease with the polar tuberculiod (TT) and lepromatous (LL) forms and the three borderlines forms including borderline tuberculoid (BT), mid borderline (BB) and borderline lepromatous (BL) [7]. TT characterized by strong cell-mediated immune response which restricts the spread of *M*. *leprae* while the LL forms are characterized by lack of cell mediated immune response which allows the growth and spread of *M*. *leprae* in these patients [8]. Studies have shown that circulating high levels of antibodies to *M*.*leprae* specific antigens in LL patients although these antibodies are unable to control the growth and the spread of *M*.*leprae* [9].

The study of the humoral immunity in leprosy has largely been restricted to antibodies. Patients towards lepromatous leprosy (LL) pole of the spectrum have higher antibody concentration as compared with the tuberculoid (TT) pole. Elevation of the polyclonal isotypes of these classes of antibody types with the highest concentration has reported in patients with LL forms compared to the other clinical types of the spectrum [10–13]. However, the role of these antibodies in the pathogenesis of leprosy is poorly understood.

Although the in-situ presences of plasma cells and B-cells have been reported in leprosy, the role of these cells in the pathology of leprosy lesions is unclear. Both Plasma cells and B-cells have been detected in tuberculoid and lepromatous leprosy lesions [14]. It was speculated that these lesional B-cells could influence T-cell responses and /or play a role in maintaining the inflammatory reaction in leprosy partly through the local secretion of antibodies. However, data supporting such hypothesis are lacking. It is generally thought that antibodies against *M*. *leprae* components do not play a significant role in protection against leprosy. However, antibodies may play a role in the uptake of *M*. *leprae* by mononuclear phagocytes and hence the pathogenesis of the diseases [15].

There are two types of leprosy reaction, type one and erythema nodosum leprosum (ENL) reactions. ENL is an immune-mediated inflammatory complication affecting about 50% of patients with lepromatous leprosy (LL) and 10% of borderline lepromatous (BL) patients [16–18]. ENL can occur before, during or after successful completion of multi-drug therapy (MDT). The onset of ENL is acute, but it may pass into a chronic phase and can be recurrent [19].

B -cells are the least studied immune cells in leprosy in general and leprosy reactions in particular. An increased percentage and absolute count of B-cells in the sera from patients with ENL has been reported [20], but normal numbers of circulating B-cells have also been reported [21]. A study looking at T-cell phenotypes in ENL lesions showed a normal proportion of B-cells in these patients [22]. In a prospective cohort study of 13 untreated patients with acute ENL reaction, polyclonal IgG1 antibody synthesis was elevated compared to patients with stable lepromatous leprosy and decreased after the disease had subsided. However, the concentration of polyclonal IgG2 had revealed the reverse trend: decreased before treatment and increased after treatment [23]. These authors also investigated the frequency of antibody secreting B-cells in the blood compartment of these patients with the Enzyme-Linked ImmunoSpot (ELISPOT) and found that the decrease in *M*. *leprae* specific IgG1 antibody was not related to the down-regulation of B-cell responses.

In addition to antibody secretion, B-cells have emerged increasingly as both effector and immunoregulatory cells in several chronic inflammatory diseases [24]. The role of B-cells in the pathogenesis of autoimmune disorders such as rheumatoid arthritis (RA) and systemic lupus erythematosus (SLE) is now being re-examined [1]. It would therefore be interesting to examine the contribution of B-cells in the pathogenesis of ENL.

## Materials and methods

### Ethics statement

Informed written consent for blood and skin biopsies were obtained from patients following approval of the study by the Institutional Ethical Committee of London School of Hygiene and Tropical Medicine, UK, (#6391), AHRI/ALERT Ethics Review Committee, Ethiopia (P032/12) and the National Research Ethics Review Committee, Ethiopia (#310/450/06). Under 18 years old patients were excluded from the study. Vulnerable and minor groups were also excluded from the study. All patient data analyzed and reported anonymously.

### Study design

A case-control study with follow-up after the initiation of prednisolone treatment was used to recruit 30 untreated patients with ENL reactions and 30 non-reactional LL patient controls between December 2014 and January 2016 at ALERT Hospital, Ethiopia.

### Patient recruitment

All patients recruited into this study were attending the ALERT Hospital, Addis Ababa, Ethiopia. The patients were classified clinically and histologically on the leprosy spectrum based on the Ridley-Jopling (RG) classification schemes [7]. ENL was clinically diagnosed when a patient with BL or LL leprosy had painful crops of tender cutaneous erythematous skin lesions [17]. New ENL was defined as the occurrence of ENL for the first time in a patient with LL or BL. Lepromatous leprosy was clinically diagnosed when a patient had widely disseminated nodular lesions with ill-defined borders and BI above 2 [19]. Patients with ENL were treated according to the World Health Organization (WHO) treatment guideline with steroids that initially consisted of 40mg oral prednisolone daily and the dose was tapered by 5mg every fortnight for 24 weeks. All patients were received WHO-recommended leprosy multidrug treatment (MDT).

### Blood sample collection and PBMC isolation

Twenty micro-litter of venous blood was collected into sterile BD heparinised vacutainer tubes (BD, Franklin, Lakes, NJ, USA) before treatment, during treatment on week 12 and after treatment on week 24 from each patient and used for PBMC isolation. PBMCs were separated by density gradient centrifugation at 800g for 25 min on Ficoll-Hypaque (Histopaque, Sigma Aldrich, UK) as described earlier [25]. Cells were washed three times in sterile 1x phosphate buffered saline (PBS, Sigma Aldrich, UK) and re-suspended with 1mL of Roswell Park Memorial Institute (RPMI medium 1640 (1x) + GlutaMAX+ Pen-Strip GBICO, Life technologies, UK). Cell viability was determined by 0.4% sterile Trypan Blue solution (Sigma Aldrich, UK) ranged from 94–98%. PBMC freezing was performed using a cold freshly prepared freezing medium composed of 20% Foetal Bovine Serum (FBS, heat inactivated, endotoxin tested ≤5 EU/ml, GIBCO Life technologies, UK), 20% dimethyl sulphoxide (DMSO) in RPMI medium 1640 (1x). Cells were kept at -80°C for 2–3 days and transferred to liquid nitrogen until use. Cell thawing was done as described [26]. The procedure is briefly described as: cells were incubated in a water bath (37°C) for 30 to 40 seconds until thawed half way and re-suspended in 10% FBS in RPMI medium 1640 (1x) (37°C) containing 1/10,000 benzonase until completely thawed, washed 2 times (5 minutes each) and counted. The percentage viability obtained was above 90%.

### Surface staining for flow cytometry

Cells were harvested, transferred to round bottomed FACS tubes (Falcon, BD, UK) and washed twice at 400g for 5 minutes at room temperature. The cells were resuspended in 50μl of PBS and incubated in 1ml of 10% human AB serum (Sigma Aldrich, UK) for 10 minutes in the dark at room temperature to block nonspecific Fc-mediated interactions, and centrifuged at 400g for 5 minutes. After resuspending cells in 50μL PBS buffer, Life/dead staining was performed at a concentration of 1μl /1mL live/dead stain (V500 Aqua, Invitrogen, Life technologies, UK) for 15 minutes at 4°C in the dark. Cells were washed once and stained for surface markers directed against CD10 (FITC), CD19 (PerCp-Cy5.5), CD27 (V500), CD21 (V450) and Isotype control (IgG1) (all from BD Biosciences, UK), Live/dead (eFluoro 780, Invitrogen, Life technologies, UK). A single-stained OneComb eBeads (affymetrix, eBioscience, UK) for all fluorescence compensation except for the live dead stain were used. For the viability dye, cells rather than beads were stained and used for fluorescence compensation.

### Sample acquisition and gating strategy

**Fig 1:**
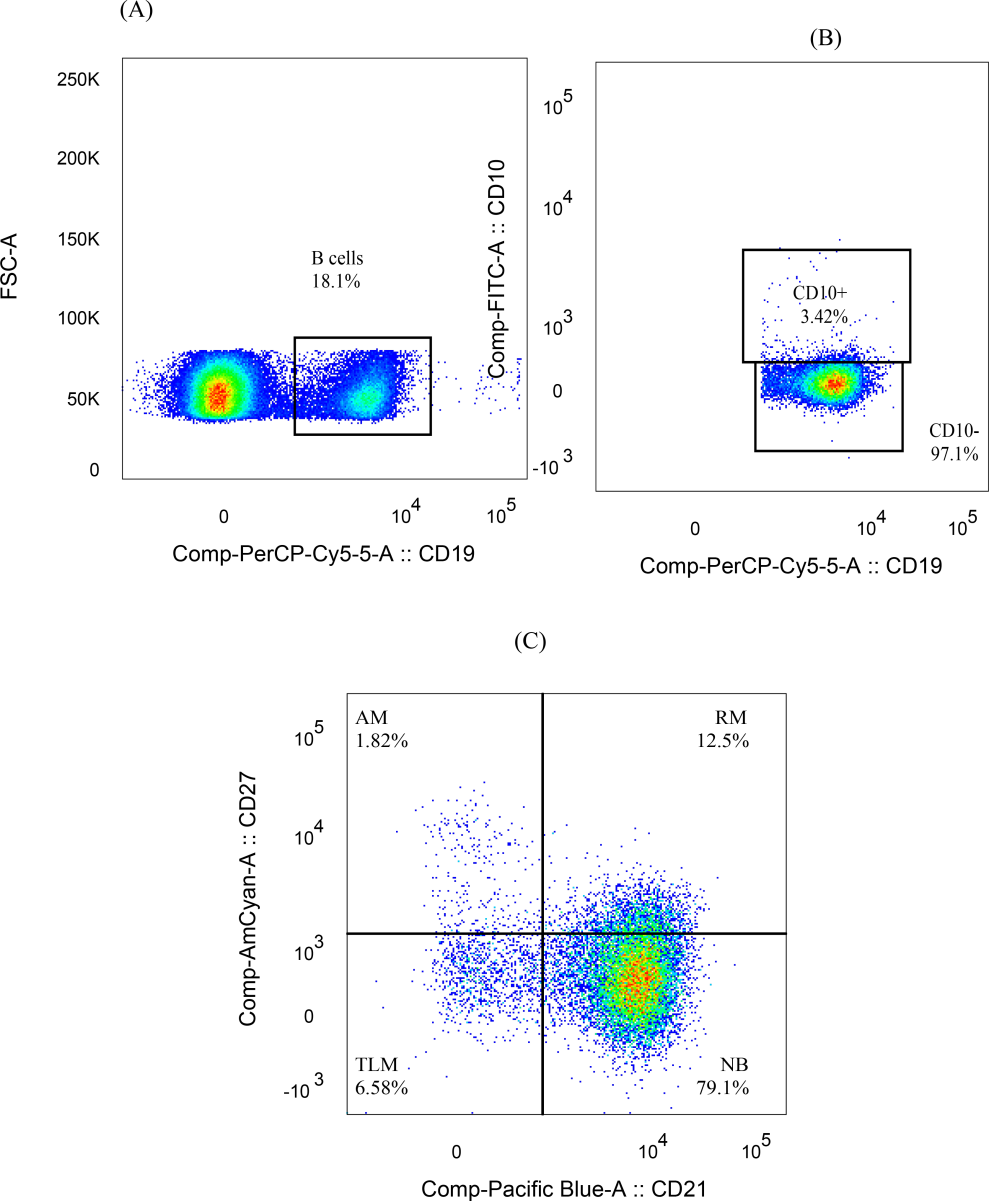
Gating strategy for B-cell sub populations; (1A) gating B-lymphocytes by FSCA versus CD19, (1B) gating mature B-cells (CD19^+^CD10^-^), (1C) memory B-cells obtained by gating mature B-cells for CD27 versus CD21.

Forward scatter height (FSC-H) versus Forward scatter area (FSC-A) plots were used to select singlets, and FSC-A versus dead cell marker plots identified viable cells. Side scatter area (SSC-A) versus FSC-A plots were used to discriminate lymphocytes from monocytes and residual granulocytes. The threshold for FSC was set to 5,000. For each sample, 500,000–1,000,000 cells were acquired. The percentage of B-lymphocytes (CD19^+^), Mature B-cells (CD19^+^CD10^-^), Nave B-cells (CD19^+^CD10^-^C27^-^CD21^+^), resting memory B- cells (CD19^+^CD10^-^CD27^+^CD21^+^), activated memory B cells(CD19^+^CD10^-^CD27^+^CD21^-)^ and tissue like memory B-cells (CD19^+^CD10^-^CD27^-^CD21^-^) was defined relative to the parent population with FlowJo version 10 (Tree Star, USA) using logicle (bi-exponential) transformation as recommended [27, 28] (Fig 1). Data were exported to Excel spreadsheet for each sample and compiled for further analysis.

### Statistical analysis

Differences in percentage of B-cell subsets were analyzed with either the two-tailed Mann-Whitney U test or the Wilcoxon signed rank non-parametric tests using STATA 14 version 2 (San Diego California USA). Graphs were produced by GraphPad Prism version 5.01 for Windows (GraphPad Software, San Diego California USA). The median and Hodges-Lehmann estimator were used for result presentation. Hodges–Lehmann is used to measure the effect size for non-parametric data [29]. P-values were corrected for multiple comparisons. The statistical significance level was set at p≤0.05.

## Results

### Total B- lymphocytes and mature B-cells

**Fig 2:**
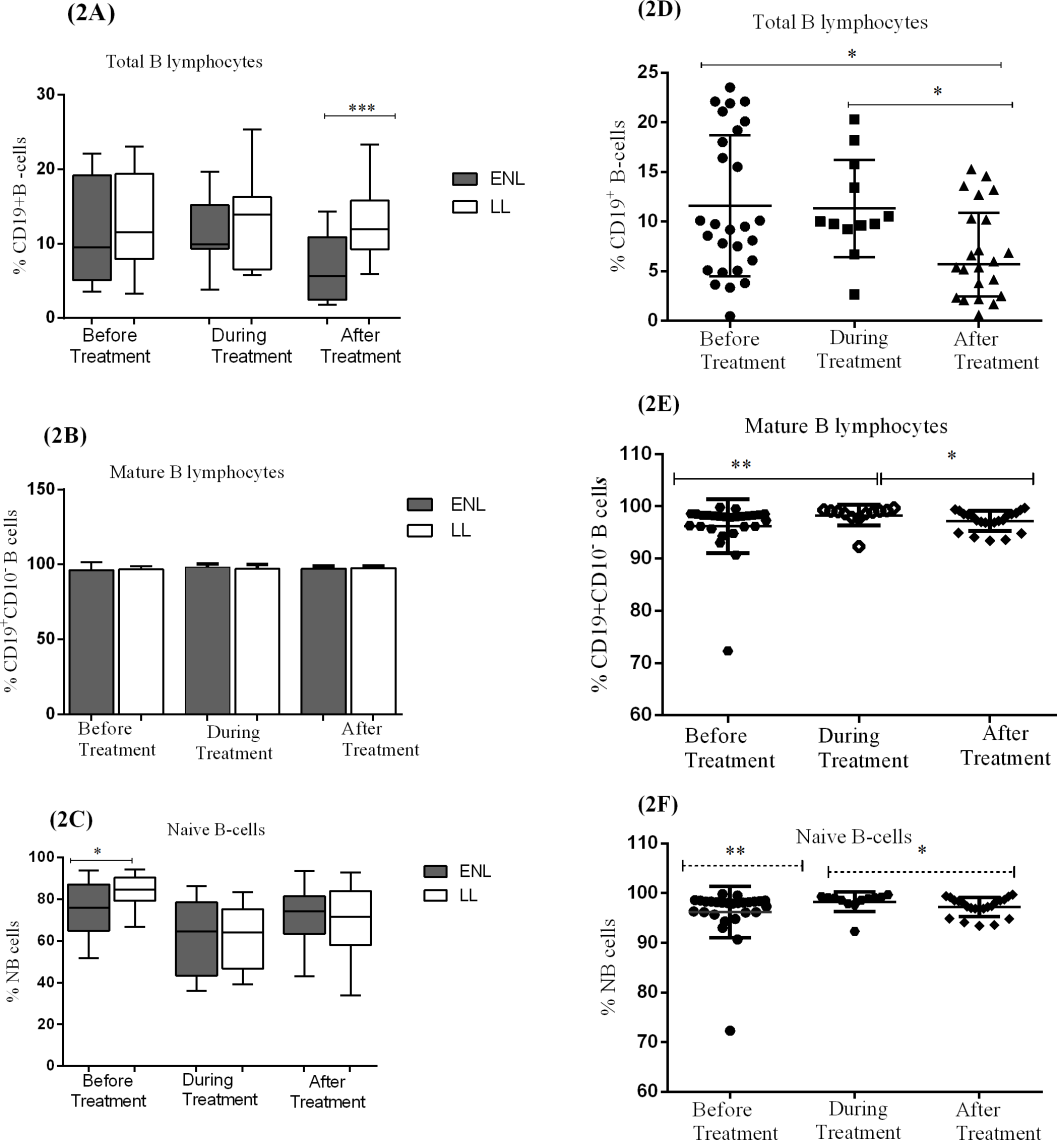
The median percentage of total B-lymphocytes (CD19^+^), mature B-cells (CD19^+^CD10^-^) and naïve B-lymphocytes (CD19^+^CD10^-^CD27^-^CD21^+^) in the PBMCs of) patients with ENL and LL controls before, during and after treatment respectively (2A,2B,2C); within ENL patients before, during and after treatment respectively (2D,2E,2F). Statistical test: Mann-Whitney unpaired test (U) (2A, 2B, 2C) and Wilcoxon matched-pairs signed rank test (2D, 2E, 2F). * *P≤ 0*.05; ** *P≤ 0*.*005;* *** *P≤ 0*.*001*. Box and whiskers show median ± interquartile range.

The median percentage of B-lymphocytes (CD19^+^) in patients with ENL (9.5%) and LL controls (11.6%) was not statistically significantly different at recruitment. During treatment, the percentage of B-cells slightly increased to 10% in patients with ENL and to 14% in LL patient controls but did not show a statistically significant difference. However, after treatment, the median percentage of B-cells appreciably decreased to 5.7% in patients with ENL while it was slightly decreased in LL patient controls to 12.0% and the difference between the two groups was statistically significantly different (P≤ 0.001; ΔHL = 6.02%) (Fig 2A).

The kinetic analysis of B-lymphocytes within ENL group at different treatment time points has shown that the median percentage of B-lymphocytes before and during treatment of patients with ENL was 9.5% and 9.9% respectively (P>0.05). However, after treatment, the percentage of these cells was significantly decreased to 5.7% compared with before and during treatment (P ≤ 0.05) (Fig 2D).

The median percentage of mature (CD19^+^CD10^-^) and immature (CD19^+^CD10^+^) B-cells in patients with ENL and LL controls before and after treatment were also measured. A significant difference was not observed with regard to the median percentage of mature and immature B-cells in patients with ENL reactions and non-reactional LL patient controls before and after treatment (Fig 2B). Similarly, the median percentage of mature B-cells before and after treatment was not statistically significantly different within ENL group (Fig 2E).

The median percentage of naive B-cells (CD19^+^CD10^-^CD27^-^CD21^+^) in untreated ENL patients was significantly lower (76.0%) than in LL patient controls (86.4%) (Fig 2C). This implies that more number of B-cells in untreated ENL patients are antigen experienced than those from LL patient controls. On the other hand, the percentage of naive B-cells within ENL group was not significantly changed before and after treatment (Fig 2F).

### Increased activated and decreased tissue like memory B-cells in untreated ENL

The median percentage of memory B-cell subtypes (resting, activated and tissue-like memory B-cells) in patients with ENL reactions and non-reactional LL controls as well as within ENL group was analysed in the unstimulated PBMCs before, during and after treatment.

**Fig 3:**
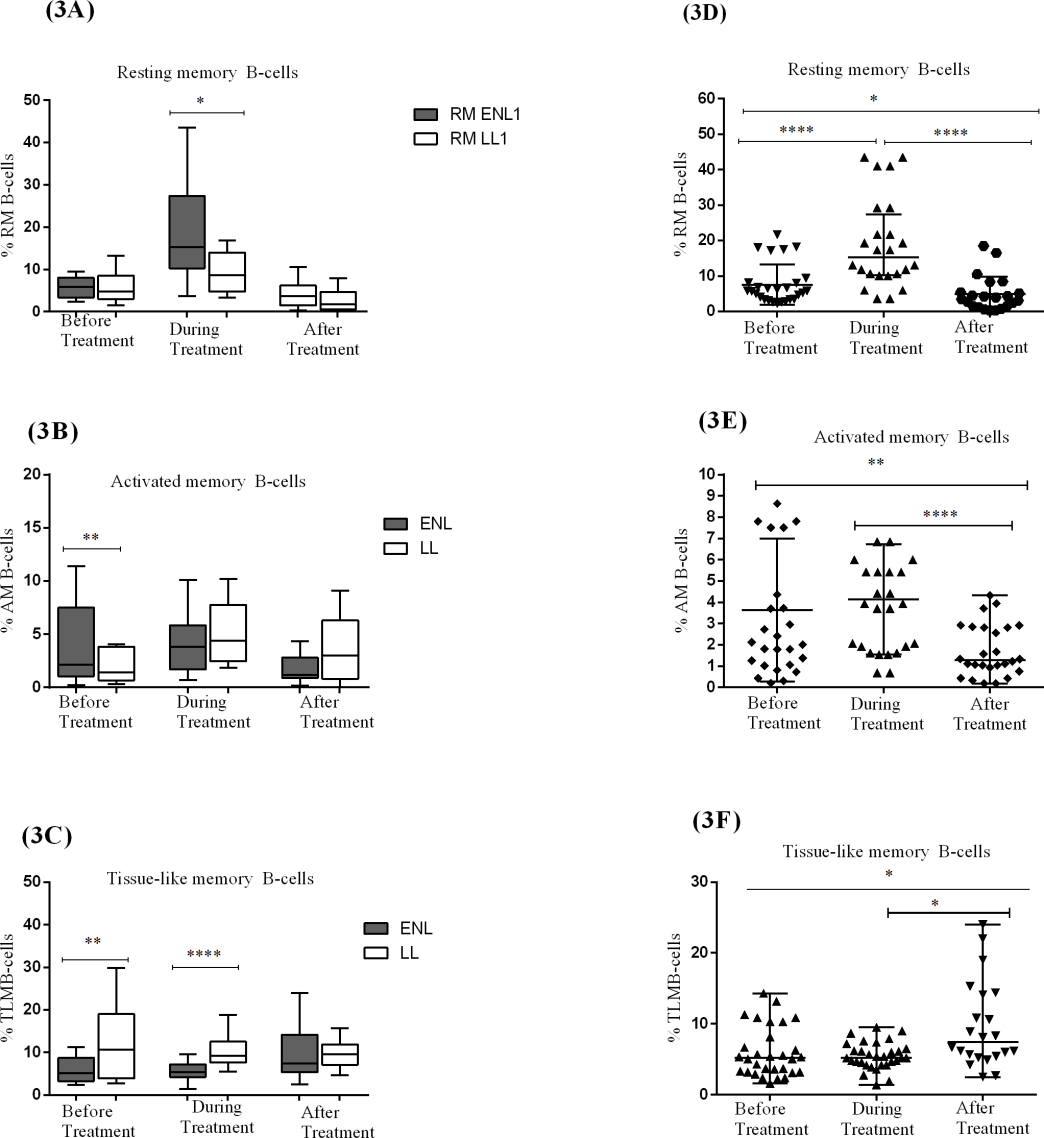
The median percentage of resting memory B-cells (CD19^+^CD10^-^CD27^+^CD21^+^), activated memory B-cells (CD19^+^CD10^-^CD27^+^CD21^-^) and tissue-like memory B-cells (CD19^+^CD10^-^CD27^-^CD21^-^) in the PBMCs of patients with ENL and LL controls before, during and after treatment respectively (3A,3B,3C); within ENL patients before, during and after treatment respectively (3D,3E,3F). Statistical test: Mann-Whitney unpaired test (U) (3A,3B, 3C)) and Wilcoxon matched-pairs signed rank test (3D, 3E, 3F)B). * *P≤ 0*.05; ** *P≤ 0*.*005;* *** *P≤ 0*.*001*. Box and whiskers show median ± interquartile range.

The median percentage of activated memory B-cells (CD19^+^ CD10^-^CD27^+^CD21^-^) was significantly higher in patients with ENL (2.6%) than in LL patient controls (1.4%) before treatment (P≤ 0.005). During treatment, the percentage of activated memory B-cells (AM) in patients with ENL and LL controls increased to 3.8% and 4.4% respectively and the difference was not statistically significantly different (P> 0.05). After treatment, the percentage of these memory cells did not change (Fig 3B).

A comparison within ENL has shown that the median percentage of activated memory B-cells in untreated ENL patients was higher (2.6%) than after treatment (1.3%) and the difference was statistically significantly different (P≤ 0.005) (Fig 3E). Hence, it seems that activated memories B-cell is associated with ENL reactions.

The median percentage of resting memory B-cells (CD19^+^ CD10^-^CD27^+^CD21^+^) was 5.8% in patients with ENL and 4.8% in LL patient controls before treatment and the result was not statistically significantly different. However, during treatment it was increased to 15.2% in patients with ENL and was higher than in LL patient controls (8.6%) (P≤0.05). After treatment, the proportion of these memory cells was decreased to below 5% in both groups and was not statistically significantly different (Fig 3A).

Analysis within ENL group has shown that the proportion of resting memory B-cells (RM) was considerably lower (5.8%) before treatment than during treatment (15.3%) (P≤0.001). After treatment, the proportion of these resting memory B-cells was decreased to 3.7% and it was significantly lower than before and during treatment (P≤ 0.05) (Fig 3D).

Interestingly, untreated ENL patients had significantly lower (5.2%) median percentage of tissue-like memory B-cells (TLM) (CD19^+^CD10^-^CD27^-^CD21^-^) than the corresponding LL patient controls (10.7%) (P≤0.05). However, after treatment a significant difference was not observed between these two groups (Fig 3C). Similarly, comparison within ENL group has shown that the median percentage of TLM is significantly lower in untreated ENL patients than after treatment (P≤ 0.05) (Fig 3F).

## Discussion

Memory B-cells are subtypes of B-cells that are formed within the germinal centres following infection. They proliferate and differentiate into antibody producing plasma cells also called effector B-cells in response to re-infection. Memory B-cells rapidly differentiate into plasmablasts that produce class-switched antibodies which are capable of clearing the infection far more quickly than naive B-cells [3]. The different classes of memory B-cells have been studied in various chronic viral infections such as hepatitis and HIV and in several autoimmune diseases [30, 31]. The role of B-cells in the pathogenesis of ENL has been speculated in several studies but has never been studied. For the first time, we studied B-cells and the memory B-cell sub-types in patients with ENL and LL controls at different time points (before, during and after treatment) to investigate the dynamics of these cells during the course of prednisolone treatment.

The percentage of total B-cells was not significantly different in the two groups before treatment. However, after treatment, the proportion of B-cells was significantly reduced from 9.5% to 5.7% in patients with ENL. The reduction of B-cells after prednisolone treatment of patients with ENL could be either transitory or associated with the subsiding of the ENL reaction which needs further investigation. The success of Rituximab to deplete B-cells for the treatment of rheumatoid arthritis has stimulated investigation of its effects in several other immune disorders, and considerable interest in the potential of drugs that can modulate B-cell function for the treatment of such diseases [32]. Thus, the finding of reduced B-cells after ENL subsides poses the question whether depleting B-cells could be effective in the treatment of ENL.

Patients with ENL had a significantly lower naïve B-cells (76.0%) than LL controls (84.6%) before treatment (P≤0.05; L-H = 6.75). It implies that more B-cells are antigen experienced in untreated ENL patients compared to in LL patient controls. However, the proportion of naïve B-cells was still unusually high in spite of the presence of abundant *M*. *leprae* antigens in these patients.

A significant difference was not observed with regard to the frequency of resting memory B cells (RM) in the two groups before treatment. However, the median percentage of activated memory B-cells (AM) was significantly higher in patients with ENL (2.6%) than in LL controls (1.4%) before treatment. Several studies have shown that activated memory B-cells are increased in patients with disease flares in systemic lupus erythematosus (SLE) [33] and rheumatoid Arthritis [34]. However, the biology of ENL and autoimmune diseases is different and whether activated memory B-cells are undesirable or not in the pathogenesis of ENL should be further investigated to arrive at a conclusive evidence. Nevertheless, activated B-cells may be primed to plasma B-cells which in turn produce immunoglobulins [30]. These immunoglobulins could interact with the *M*.*leprae* antigen and thereby form excess immune-complexes beyond clearance or activated B-cells may serve as antigen presenting cells i.e. presenting *M*.*leprae* antigens to T-cells. Depending on the magnitude of antigen presentation, the T-cell response could be excessive and may cause tissue damage.

Patients with ENL had lower percentage of tissue-like memory (TLM) B-cells (5.2%) than LL controls (10.7%) before treatment. Several studies have indicated that TLM B-cells represent the exhausted state of B-cells since they express several inhibitory receptors, including the immunoreceptor tyrosine-based inhibitory motif (ITIM)-containing inhibitory receptor Fc receptor-like protein 4 (FcRL4) [35]. TLM B-cells show a reduced tendency to proliferate in response to cognate antigen [36]. The expression of FcRL4 on human B-cell lines disrupts immune synapse formation and blocks antigen induced B-cell receptor (BCR) signalling [37]. They also express not only FcRL4 but also a number of other inhibitory and chemokine receptors that would reduce the likelihood of B and T cell interaction [5]. It has been shown that a specific siRNA knockdown of FcRL4 and other inhibitory receptors may lead to a rescue of Ig secretion and proliferation in these tissue-like memory B-cells [38]. In contrary to our findings, increased proportion of TLM have been reported in chronic infections such as in hepatitis C virus and malaria infections, and in certain autoimmune diseases [39, 40]. The decreasing tendency of TLM and increased secretion of activated memory B-cells may indicate the activation of B-cells when LL patients develop ENL reactions. The finding of increased percentage of TLM in LL patients in this study may partly explain why B-cells are unable to control *M*.*leprae* multiplication in spite of their abundance in LL patients. The T-cell unresponsiveness in LL may also be associated with the increased production of TLM B-cells. It appears that the higher frequency of TLM B-cells in LL could alter the B-cell/T-cell interaction through blocking B-cell receptors and this hypothesis could be a fertile area for future investigation. Once, this hypothesis is proved, the search for the B-cell immunomodulators that safely overcome this exhaustion phenotype may be necessary in order to develop proper immune response to LL patients. On the other hand, the significant reduction of TLM during ENL reaction suggests the down regulation of inhibitory molecules and thereby increases immune activation in LL patients leading to the onset ENL reaction. Thus, our finding implies that TLM B-cells could have a role in the initiation of ENL reactions which is a new exciting area for further investigation.
